# UPLC-HRMS-Based Plasma Metabolomic Profiling of Novel Biomarkers by Treatment with KDZI in Cerebral Ischemia Reperfusion Rats

**DOI:** 10.3390/molecules23061315

**Published:** 2018-05-30

**Authors:** Chunguo Wang, Chenyue Liu, Min Wang, Quantao Ma, Yaqi Li, Ting Wang, Baosheng Zhao

**Affiliations:** 1 Beijing Research Institute of Chinese Medicine, Beijing University of Chinese Medicine, Beijing 100029, China; wangcg1119@126.com; 2 School of Chinese Material Medica, Beijing University of Chinese Medicine, Beijing 100029, China; liuchenyue633@163.com (C.L.); weme9916@163.com (M.W.); maquantao@bucm.edu.cn (Q.M.); liyaqi@bucm.edu.com (Y.L.); 3 School of Traditional Chinese Medicine, Beijing University of Chinese Medicine, Beijing 100029, China

**Keywords:** Kudiezi injection, UPLC-HRMS, middle cerebral artery occlusion and reperfusion

## Abstract

Kudiezi injection (KDZI), also known as Diemailing injection, is a traditional Chinese medicine injection of the composite plant *Ixeris sonchifolia* Hance (also known as Kudiezi), and has been widely used to treat coronary heart disease, angina pectoris, and cerebral infarction, but its pharmacological mechanisms remain unclear. This study is designed to explore the effects of KDZI on middle cerebral artery occlusion and reperfusion (MCAO/R) rats, and to identify metabolic features of cerebral ischemia reperfusion by using a nontargeted metabolic profiling method based on ultra-performance liquid chromatography-high resolution mass spectrometry (UPLC-HRMS). In this process, 32 potential biomarkers were found in plasma. KDZI significantly upregulated the levels of taurochenodesoxycholic acid, leucine, l-phenylalanine, l-tryptophan, arachidonic acid (ARA), and phosphatidyl ethanolamines (PE), phosphatidyl cholines (PC) and downregulated the levels of l-valine and 5-hydroxyindole-3-acetic acid (5-HIAA) in plasma. The results indicated that the mechanisms of KDZI on MCAO/R were related to the mechanisms of amino acid and lipid metabolism.

## 1. Introduction

The process of aging has hastened the increasing number of elderly people in human society. In the meantime, the risk of cerebrovascular disease increases year by year. Among cerebrovascular disease, ischemic cerebrovascular disease is one of the diseases with the highest morbidity, which has the features of high incidence, high morbidity, and high mortality [1]. Cerebral ischemic infarction can cause disability, cognitive dysfunction, vascular dementia, and even death. The increasing morbidity and mortality of cerebral ischemia seriously affects the physical and mental health of the elderly, as well as an unbearable financial toll for both society and families [2].

When ischemic cerebrovascular disease attacks, the priority goal is to recover the blood supply of the brain as soon as possible to avoid brain tissue necrosis. However, if the treatment is not timely, cerebral ischemia reperfusion injury will occur. Therefore, ischemic cerebrovascular patients should be evaluated rapidly in the early phase to receive timely and appropriate treatment. Unfortunately, the diagnostic features of the ischemic cerebrovascular are still limited. Typical diagnosis relies upon medical equipment, such as computed tomography (CT) or magnetic resonance imaging (MRI), but these examinations are time-consuming and costly. Therefore, it is necessary to develop novel biomarkers through blood metabolomics for the diagnosis and prognosis of ischemic cerebrovascular [3].

Till now, the mechanisms for ischemic cerebrovascular have been dramatically elucidated and considered a series of complex pathophysiological processes, including energy metabolism disorder, toxicity of excitatory amino acids, overloaded intracellular calcium, increase of oxygen free radicals, endothelial cells injury, apoptosis gene activation and inflammatory response [4,5,6,7], but the metabolite profiling of ischemic cerebrovascular disease remains unknown. Therefore, the study of the metabolic variations might be expected to provide new insights into the pathogenesis of ischemic cerebrovascular disease.

The other major challenge for ischemic cerebrovascular disease is that no effective and generally accepted medicine is available except for tissue-type plasminogen activator (t-PA ) [8]. Unfortunately, the clinical application of t-PA is limited because of the symptomatic intracerebral hemorrhage adverse reaction and narrow therapeutic window. Increasing amounts of evidence in treating ischemic cerebrovascular disease demonstrate that treatment regimens containing multiple drugs lead to maximal therapeutic efficacy with minimal adverse effects. Therefore, combination therapy becomes a rational and promising choice, and has been advocated in traditional Chinese medicine (TCM) to prevent and cure ischemic cerebrovascular disease for thousands of years. Varieties of herbal formulae or extracts have been shown to exhibit anti-cerebral ischemic effects, and Kudiezi injection (KDZI) is one of the most representative ones. KDZI, also known as Diemailing injection, is a TCM injection of the composite plant *Ixeris sonchifolia Hance* (also known as Kudiezi) [9]. KDZI contains flavonoids, adenosine, sesquiterpene lactones, and triterpenoid saponins, etc. [10,11]. Studies have shown that KDZI can inhibit thrombosis, increase fibrinolytic activity, dilate blood vessels, reduce vascular resistance, and increase brain and blood flow [12]. KDZI also has protective effect on brain injury in rats with cerebral ischemia reperfusion, but the mechanism is not clear yet [13,14,15,16]. Systemic studies are, therefore, required to provide a comprehensive evaluation of the efficacy of KDZI.

Metabolomics, an important part of systemic biology, was initially defined by professor Nicholson et al. [17]. Metabolomics aims at comprehensive characterization of the total metabolome in a biological system and the responses that dynamic metabolomics response to alterations of inner and external factors [18,19]. In recent years, metabolomics has been widely used in the diagnosis of heart disease [20], diabetes mellitus [21], and cancer [22]. However, the plasma metabolic profiling of ischemic cerebrovascular remains poorly understood and no metabolomic assessment of the anti-cerebral ischemia effects of KDZI is published.

In this study, a middle cerebral artery occlusion reperfusion (MCAO/R) rat model was established to mimic ischemic cerebrovascular, and plasma samples were collected from the MCAO/R rat model, controls, sham surgery group, and KDZI-treated group, an ultra-performance liquid chromatography-high resolution mass spectrometry (UPLC-HRMS)-based metabolomics approach was developed to characterize the metabolic biomarkers on ischemic cerebrovascular, and to discover potential targets of KDZI on ischemic cerebrovascular treatment. Partial least squares-discriminant analysis (PLS-DA) was further utilized to identify the ischemic cerebrovascular-related metabolic alterations and investigate the anti-cerebral ischemia mechanisms of KDZI. In addition, the study provided a reasonable basis for clinical treatment programs and a scientific example to highlight the power of the LC-HRMS system for sophisticated biological datasets.

## 2. Results

### 2.1. Effects of KDZI on Pathological Brain Morphology Alterations in MCAO/R Rats

**Figure 1:**
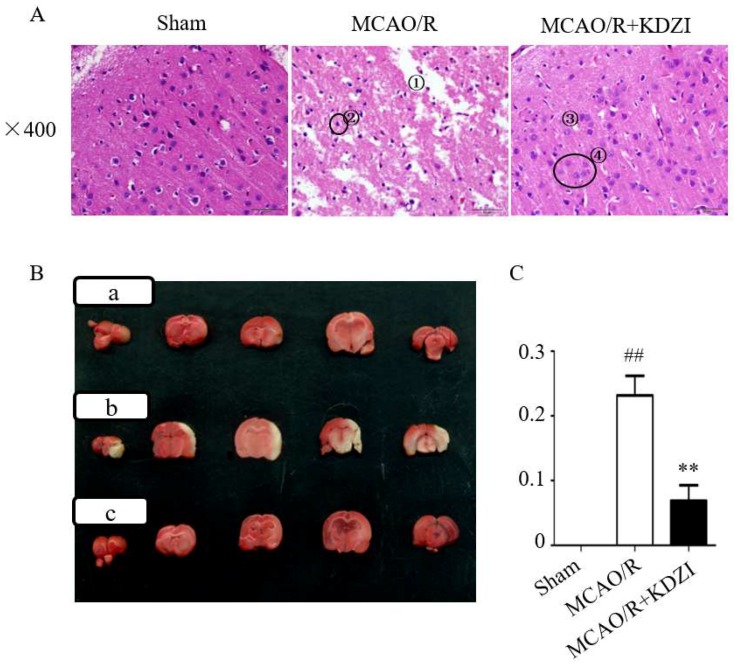
The representative images of HE (**A**) and TTC (**B**,**C**) stainings of brain sections showing the neuroprotective effect of KDZI on focal cerebral ischemia. The place marked by ① in Figure 1A indicates cytoclasis and ② indicates the karyopyknosis of the cortex of the brains in MCAO/R rats. ③ indicates brain edema was significantly alleviated and ④ indicates the neuronal cells almost returned to normal. Figure 1B shows: (**a**) the sham group; (**b**) MCAO/R group; and (**c**) MCAO/R + KDZI group. Data are presented as mean ± SD. ^##^ *p* < 0.05 with the sham group, ** *p* < 0.05 compared with MCAO/R group.

Hematoxylin and eosin (HE) staining (Figure 1A) showed that no neuronal damage or inflammatory cell infiltration was observed in the cerebral cortex of rats in Sham group, the number and morphology of neuronal cells were normal, and the cell membrane and nucleolus were clear. However, the ischemic cortex of the brains in MCAO/R rats displayed cytoclasis, karyopyknosis, and neuronal cells were reduced. After seven days of KDZI administration, brain edema was significantly alleviated, neuronal cells almost returned to normal, and the structure became clear and integral.

### 2.2. Effects of KDZI on Infarct Size in MCAO/R Model Rats

As shown in Figure 1B,C, the results of 2,3,5-triphenyltetrazolium chloride (TTC) staining demonstrated that the relative area ratio of the infarcted area of rats in the model group was 0.232, which was statistically significant (compared with rats in the sham group, *p* < 0.01), indicating that the MCAO/R model was successfully established. Compared with the MCAO/R group, the relative ratio of the infarcted area of rats in the KDZI administration group was 0.108, indicating that the KDZI significantly reduced the infarct size. The results indicated that KDZI can significantly reduce the cerebral infarction size and ameliorate cerebral ischemia.

### 2.3. Metabolic Profiling of Plasma Sample

To analyze the profile of the metabolites, the signal responses of plasma metabolites in ESI^+^ and ESI^-^ modes were combined. Both ESI^+^ and ESI^-^ displayed obvious differences in the metabolite profile in the plasma samples, indicating that the combination of positive and negative modes can be used to analyze plasma metabolism to a certain degree.

**Figure 2:**
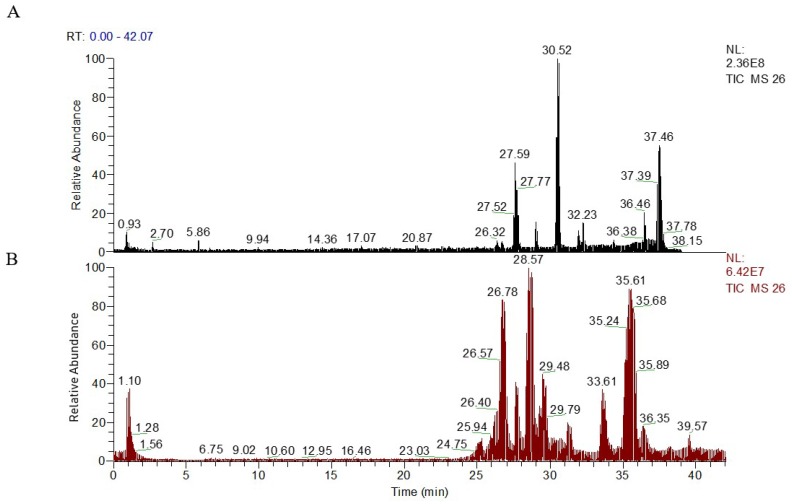
Representative UPLC-HR-MS total ion chromatograms (TICs) of serum samples from ESI^+^ model (**A**), intensity of TIC = 2.36 × 10^8^; and ESI^−^ model (**B**), intensity of TIC = 6.42 × 10^7^.

The quality control (QC) samples were used in the optimization of separation and determination conditions. The typical base peak intensity chromatograms of plasma samples, obtained in ESI^+^ and ESI^-^ under the optimized conditions, are presented in Figure 2. Some distinctly different peaks between the sham group and the ischemia model group were found in the base peak intensity chromatograms and were then highlighted. The precision and repeatability of the system were evaluated by the reduplicate analysis of six injections of the same QC sample. The relative standard deviation (*RSD*) of retention times (t_R_) and peak intensities for the selected ions from QC samples were calculated to evaluate the method performance.

As shown in Table 1, the *RSD* of the retention times for precision, stability and repeatability were 0.035–0.25%, 0.09–1.88%, and 0.04~0.59% in positive ion mode and 0.03–0.65%, 0.07–0.56%, and 0.08–0.56% in negative ion mode; the *RSD* of the peak intensities for precision, stability, and repeatability were 3.41–7.10%, 3.72–7.70%, and 4.52–6.98% in positive ion mode and 2.91–7.33%, 2.30–7.07%, and 3.45–8.33% in negative ion mode. The results of the precision, stability, and repeatability demonstrates that the proposed method is a robust method and the analysis in the study is satisfied.

### 2.4. Multivariate Data Analysis of UPLC-LTQ/Orbitrap Data

**Figure 3:**
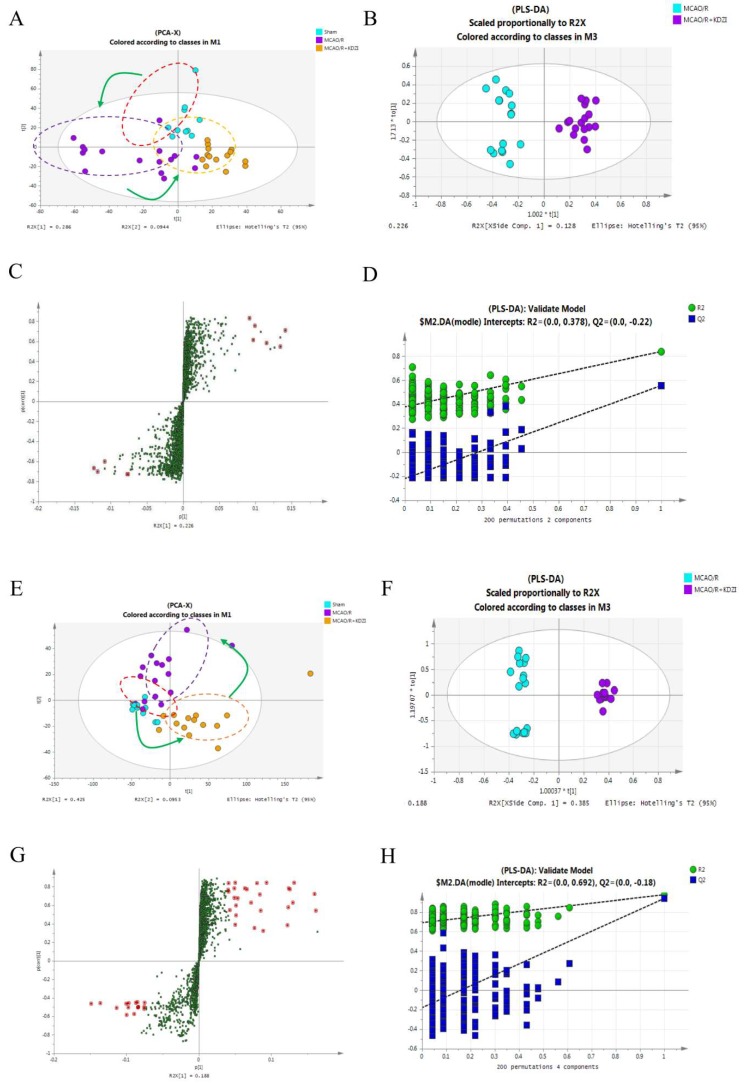
Multivariate data analysis of LC-MS in plasma obtained from sham, MCAO/R, and MCAO/R + KDZI group rats. (**A**,**E**) are principal component analysis (PCA) score plots for the components indicating the separation among the three groups; (**B**,**F**) are score scatter plots generated from partial least squares discriminate analysis (PLS-DA); (**C**,**G**) are S-plot of PLS-DA explaining the separation of (**A**,**E**) (variable importance in the projection(VIP) > 1.0 have been marked); and (**D**,**H**) are the statistical validation of the PLS-DA model by permutation testing. (**A**–**D**) were under positive ion mode; and (**E**–**H**) were under negative ions mode.

TTC and HE stainings showed that the injury of cerebral ischemia-reperfusion of rats with treatment of KDZI was significantly improved compared with that of the MCAO/R group, so we used non-targeted LC-MS to detect the anti-ischemia mechanism of KDZI. Firstly, based on the ANOVA test, we found a total of 449 ions changed significantly (*p* < 0.05) between MCAO/R and sham groups. The ions with statistical significance were merged and introduced into SIMCA-P for multivariate analyses. According to the PCA model shown in Figure 3A,E, there was a good separation among sham, MCAO/R, and KDZI groups, the R2X(cum) value were 0.71 in ESI^+^ and 0.647 in ESI^−^ mode, respectively, which indicated that acute ischemia reperfusion and the intervention of KDZI can cause significant changes in the related components.

In order to examine the possible influence on the metabolic pattern of KDZI, and to verify the metabolic features of MCAO/R, the supervised method PLS-DA was used to isolate the variables that were responsible for differences among three groups. In ESI^+^ mode, R2X(cum) = 0.797, R2Y(cum) = 0.989; in ESI^−^ mode, R2X(cum) = 0.657, and R2Y(cum) = 0.949 (Figure 3B,F). The loading S-plot shown in Figure 3C,G explains the separation above and is used to discover biomarkers between the three groups. Furthermore, the robustness of the PLS-DA models was assessed by a 200-times permutation test. No over-fitting (Q2Y > 0.5) was observed according to the results of the permutation test (Figure 3D,H) [23].

### 2.5. Identification of Metabolites

As illustrated by PLS-DA plot, 210 ions were selected (VIP> 1) for chemical structure identification, which contributed to the good separation of sham, MCAO/R, and KDZI rats. Based on the published strategy [24,25], the following steps were performed for the identification of chemical structures in this study. The quasi-molecular ions were confirmed and the exact masses of the monoisotopic molecular weights were used to search reliable online databases such as the Human Metabolome Database [26] and Metlin [27]. The MS/MS spectra were analyzed to confirm the structure of the identified metabolites by the Mass Bank [28] and Lipidbank [29]. Identification of MS/MS using the mzCloud database, and the structures of the compounds were identified through manual comparison. Compound Discoverer 2.0 software (ThermoFisher Scientific Inc, USA) matches the mzCloud spectra of MS^1^ and MS^2^ mass spectrum data with accurate molecular weight. The parameters are MS^1^ mass accuracy <5 ppm and the library search algorithm is HighChem Low + High Res. The score matching threshold is greater than, or equal to, 60 (the higher the matching is, the more accurate it is 100). During the search process, isotope matching and deduction of background noise (S/N ratio < 10,000 is cutoff) are performed.

Subsequently, 32 metabolites were identified. The names of the biomarkers, the related enzymes and the related metabolic pathways are presented in Table 2. Among them, the levels of taurochenodesoxycholic acid, leucine, l-phenylalanine, l-tryptophan, arachidonic acid, and PE were significantly decreased in the MCAO/R group, compared with the sham group, after seven days of KDZI administration, the compounds mentioned above were increased. In contrast, the level of l-valine and 5-hydroxyindole-3-acetic acid (5-HIAA) in the plasma of the MCAO/R group were increased, and KDZI decreased the contents of l-valine and 5-HIAA in plasma (*p* < 0.05).

**Figure 4:**
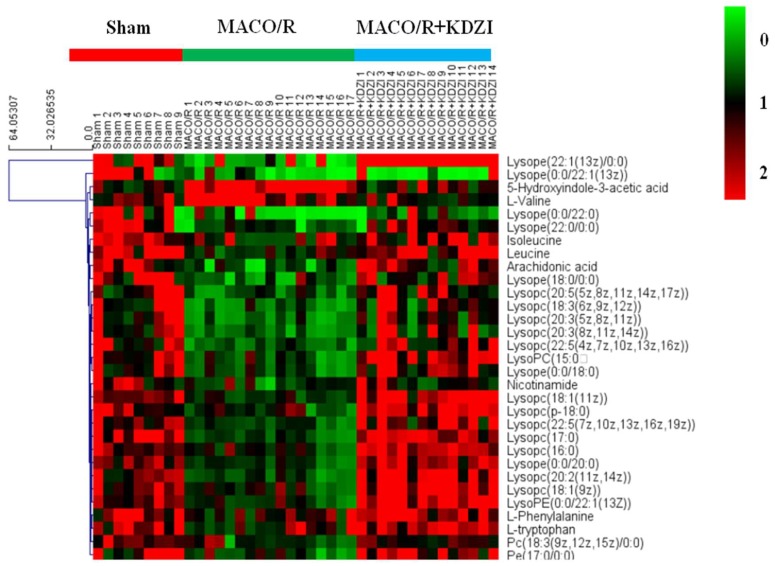
Heatmap of the 32 identified metabolites showing a significant difference among samples from the sham, MCAO/R, and MCAO/R + KDZI groups constructed by using by using the software Multi Experiment Viewer (MeV) v4.8. Rows: potential biomarkers; columns: samples. The color key indicates the relative amounts of metabolites: Light green represents the lowest; light red represents the highest.

In addition, the hierarchical cluster analysis was performed to assess the relatedness and the distance of samples in different groups, and the heatmap was presented as a visual aid by using those biomarkers as variables. On the y-axis, the cluster was broken into three parts, including the sham group, MCAO/R group, and MCAO + KDZI (Figure 4).

### 2.6. Structure Identification

**Figure 5:**
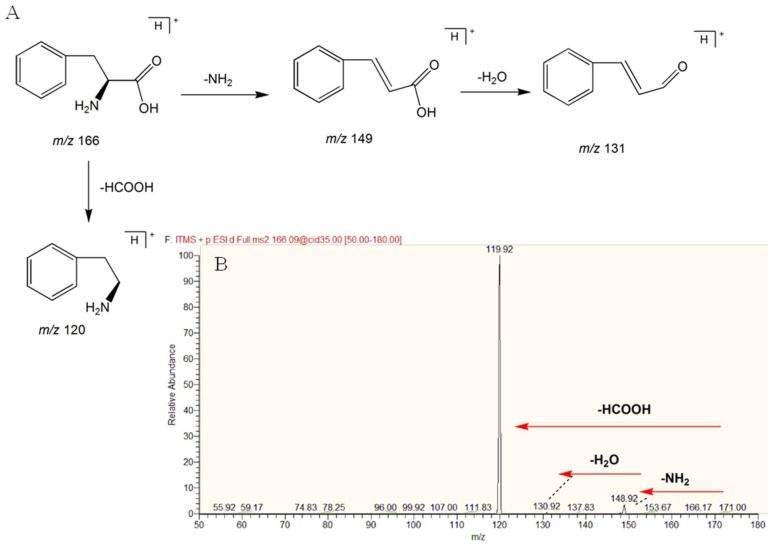
(**A**) Fragmentation pathway and product ion spectrum of L-phenylalanine; (**B**) MS/MS spectrum of l-phenylalanine by mzCloud.

In this study, a total of 32 compounds were identified in rats’ plasma. l-phenylalanine was used as an example to elucidate its structure. In HESI positive mode, a [M + H]^+^ peak could be observed, the retention time of l-phenylalanine was 2.829 min, and the *m*/*z* was 166.08665 (mass error −2.4 ppm). Fragment ions of *m*/*z* 148.92, *m*/*z* 130.92, *m*/*z* 119.92, and *m*/*z* 117 appeared in MS^2^ by LTQ-Orbitrap mass. The peak of *m*/*z* 119.92 appeared in the form of base peak in the MS^2^ spectrum, which differed from the peak of the excimer ion by 46 Da and one molecule of formic acid, so it was presumed to be a carboxylic acid compound. In addition, both the excimer ions and the main fragments were even numbers. The compound is presumed to be a nitrogen-containing compound. Additionally, *m*/*z* 148.92 was a fragment ion of an excimer ion-missing NH_2_, which was identified as phenylalanine by comparison with databases, such as Metlin and HMDB. The mass spectrogram and the cleavage behavior are shown in Figure 5A,B. The identifications of other compounds are the same as above.

### 2.7. Metabolic Pathway Analysis

**Figure 6:**
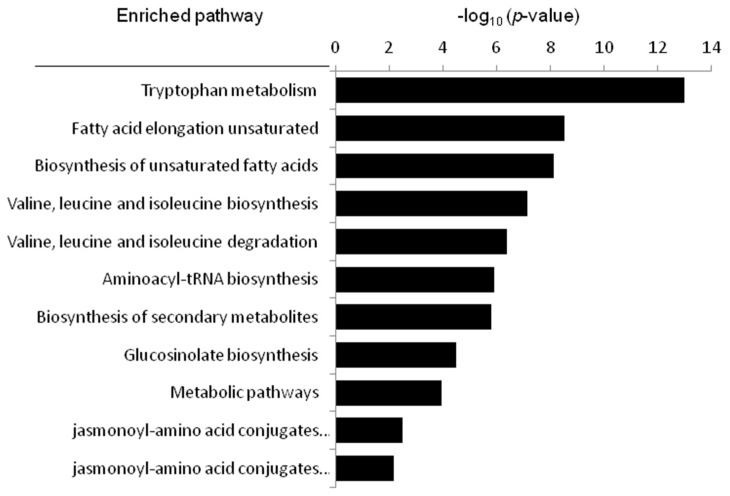
The metabolic pathways related to cerebral ischemia-reperfusion, as analyzed by MetaboAnalyst.

On the basis of the identified compounds and the Kyoto Encyclopedia of Genes and Genomes online database of metabolic pathways [30], a map of metabolic pathways of the treatment of cerebral ischemia-reperfusion injury with KDZI was constructed, shown in Figure 6 and Table 3. The relevant metabolic pathways and corresponding *p*-values and *FDR* correction (FDR correction value of the *p*-value) are shown in Table 3 and Figure 6. Among them, tryptophan metabolism, valine, leucine, and isoleucine degradation, and valine, leucine, and isoleucine biosynthesis were the major relevant metabolic pathways. Tryptophan, valine, leucine, and isoleucine were important factors related to cerebral ischemia.

## 3. Discussion

### 3.1. Characterized Potential Biomarkers

Cerebral ischemic infarction can cause disability, cognitive dysfunction, vascular dementia and even death. The increasing morbidity and mortality of cerebral ischemia seriously affects the physical and mental health of the elderly, as well as delivering an unbearable financial toll on society and families. Therefore, it is better to find serum biomarkers as compared to physical diagnosis, such as CT, as early as possible before the ischemic injury. Hence, we designed this study to find biomarkers of cerebral ischemia reperfusion by using metabolomics technology. In this research, we found that KDZI had the effect of reducing embolism area of MCAO/R rats and regulating of metabolites relevant to cerebral ischemia reperfusion. 32 potential biomarkers were found in plasma in the process. To date, metabolites of ischemic cerebral reperfusion rats with the treatment of Kudiezi injection have been analyzed using UHPLC-LTQ-Orbitrap, but only 15 metabolites were found in their study [31].

We observed taurochenodesoxycholic acid (TCDCA) were increased after KDZI administration to MCAO/R rats. In the studies of excitatory neurotoxicity and hypoxia, TCDCA can maintain the function of mitochondria, decrease the calcium influx triggered by glutamate neurotoxicity, and protect nerve function. Taurine interacts with gamma-aminobutyric acid (GABA) to activate GABA receptors and glycine receptors, enhance the neuroprotective effects, assist in the transport of ions, like Ca^2+^, Mg^2+^, Na^+^, and K^+^, and to maintain normal osmotic pressure intracellularly and extracellularly. The decreased level of taurine indicated that brain tissues of cerebral ischemia and reperfusion rats produce excitotoxic neurotoxicity. Hua Liu et al. [32] found that taurine had the effect of an anti-free radical on cerebral ischemic reperfusion damage. Zhong Yang et al. [33]. demonstrated that TCDCA had the effects of downregulating the level of malondialdehyde (MDA) and upregulating the level of glutathione peroxidase (GSH-Px) in MCAO/R rats.

**Figure 7:**
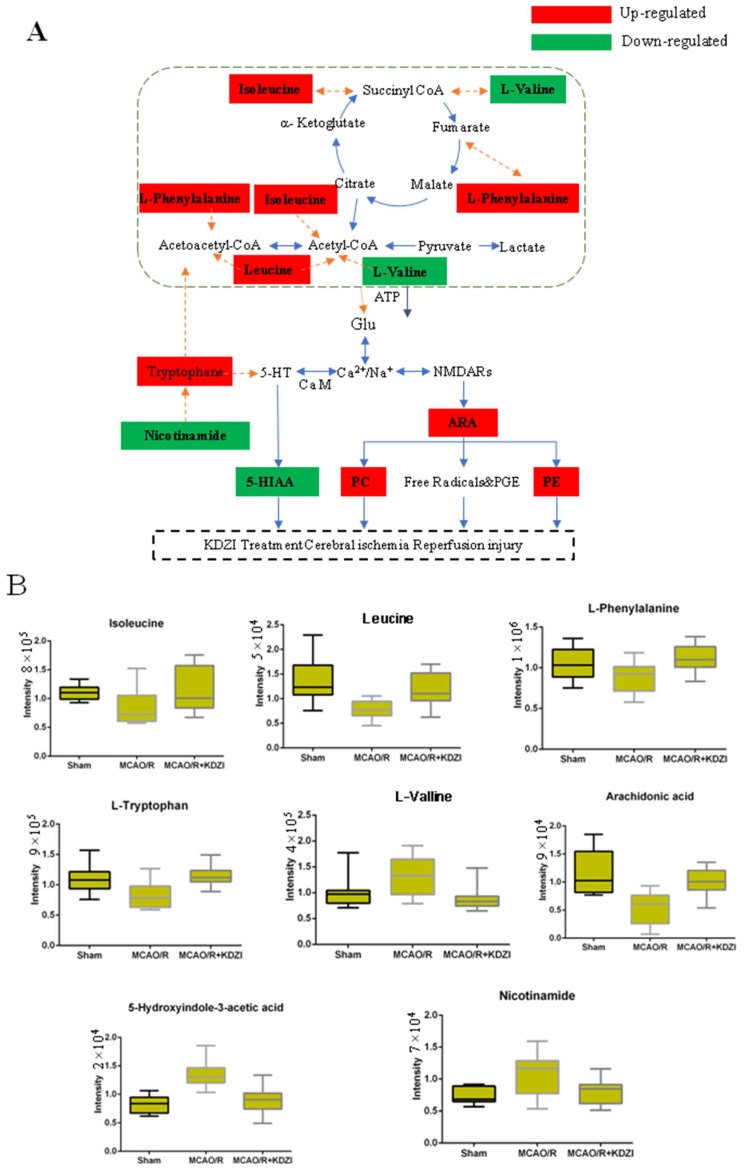
(**A**) Possible mechanism of anti-cerebral ischaemia reperfusion effect KDZI. (**B**) Typical metabolite variations in plasma samples from sham, MCAO/R, and MCAO/R + KDZI. Glu, glutamate; CoA, acetyl coenzyme A; ARA, arachidonate; 5-HT, 5-hydroxytryptamine; CAM, calmodulin; PE and PC, phospholipid; NMDARs, *N*-methyl-D-aspartic acid receptors.

Our results showed that the level of l-valine was decreased in MCAO/R rats and the levels of isoleucine, l-leucine, l-phenylalanine, and L-tryptophan were increased with the treatment of KDZI. Similarly, Liu Siyi et al. [34] also found the biosynthesis of phenylalanine and tryptophan in rats with the treatment of Kudiezi injection. These amino acids enter the tricarboxylic acid cycle by deamination or transamination and promote the production of adenosinetriphosphate (ATP). Changes of these amino acid levels cause the reduction of ATP provided by tricarboxylic acid cycle. Brain cells are highly dependent on mitochondrial energy. Thus, the decrease of ATP provided by the tricarboxylic acid cycle can cause brain ischemia. When cerebral ischemia occurs, oxygen cannot be adequately supplied, the brain tissues of rats are mainly supplied by anaerobic glycolysis, and amino acid contents are significantly reduced. Interestingly, these amino acids can be regulated by the treatment of KDZI. As depicted in Figure 7B, the levels of isoleucine, l-leucine, l-phenylalanine, and l-tryptophan were significantly decreased, and the level of l-valine was increased in the rats of the MCAO/R group compared with that of the sham group; with the treatment of KDZI, the levels of isoleucine, l-leucine, l-phenylalanine, and l-tryptophan were significantly increased, and the level of l-valine was decreased. 

After cerebral ischemia, a large number of free radicals are produced through arachidonic acid (ARA) metabolism and other ways, and these free radicals have neurotoxic actions on intracellular proteins, lipids, and nucleotides, resulting in neurocyte damage. The neurotoxic effects of oxygen radicals can be summarized as follows: (1) they act on polyunsaturated fatty acids, and cause lipid peroxidation; and (2) they induce the crosslink of macromolecules, such as DNA, RNA, polysaccharides, and amino acids, which result in activity reduction/loss of the large molecules. As demonstrated in Figure 7B, the level of ARA was significantly decreased in the rats of the MCAO/R group compared with that of the sham group; with the treatment of KDZI, the level of ARA was significantly increased.

Cerebral ischemia produces a large number of free radicals, and these free radicals attack nerve membranes and blood vessels that are rich in polyunsaturated fatty acids and produce lipid peroxides and hydroperoxides. The loss of the unsaturated fatty acids destruct the integrity of cellular structures. Meanwhile, the membrane permeability, ion transport, and membrane screen function are also severely affected, resulting in cell necrosis. Similarly, we found that the level of ARA in rats of MCAO/R + KDZI group increased significantly.

Twenty-two phosphatidyl ethanolamines (PE), phosphatidyl cholines (PC) were changed in MCAO/R rats compared with the rats in Sham group. Both PE and PC were increased by treating MCAO/R with KDZI. Lecithin can be hydrolyzed to produce glycerol phosphorylcholine (GPC), phosphorylcholine (Pcho), and choline, the phosphorylcholine is hydrolyzed into choline with the catalysis of alkaline phosphatase; at the same time, choline produces phosphorylcholine in an ATP-containing environment and then synthesizes lecithin. The studies of Djuricic et al. [23] showed that, in the case of cerebral ischemia, the content of choline increased with the decreased of level of ATP. Kozuka et al. [35] found that, in the early stage of cerebral ischemia in spontaneous hypertensive rats, the level of choline increased at 1–2 h. The studies showed that, three hours after cerebral ischemia-reperfusion in rats, the membrane phospholipids were largely hydrolyzed. Meanwhile, because of the decrease of ATP in the brain tissues, and the reduction of efficiency of choline synthesizing lecithin, the content of choline saw a significant increase in the left-brain tissues of MCAO/R rats, and the level of phosphorylcholine decreased.

### 3.2. Possible Mechanism of Anti-Cerebral Ischaemia Reperfusion Effect KDZI

The profile of metabolic network including the 32 significantly changed metabolites in plasma were shown in Figure 7A, B. This anti-cerebral ischemia reperfusion mechanism of KDZI can be described as below: KDZI has the effect on regulating the levels of l-valine, isoleucine, l-phenylalanine, and leucine to enter the tricarboxylic acid cycle by deamination or transamination and promote the production of ATP to provide energy for the brain and anti-cerebral ischemia. KDZI can increase ARA by regulating calcium and sodium ions and NMDARs, resulting in elevated PE, PC, and improved cerebral ischemia. On the other hand, glutamate affects 5-HT secretion, KDZI can decrease 5-HIAA secretion and improve cerebral ischemia.

## 4. Materials and Methods

### 4.1. Chemicals and Reagents

Kudiezi injection (KDZI, Lot No. 140506, Jilin Tonghua Huaxia Pharmaceutical Co., Ltd., Tonghua, China), acetonitrile, methanol, and formic acid (HPLC grade) were purchased from Thermo Scientific (Waltham, MA, USA). Trypsin (mass spectrometry grade) was from Promega (Madison, WI, USA). Double distilled water (18.2 MΩ·cm) was from a Milli-Q system (Millipore, Bedford, MA, USA).

### 4.2. Animals and Grouping

Male Sprague Dawley rats (220 g ± 20 g) were purchased from Beijing SiBeiFu Animal Technology Co. Ltd. (Beijing, China; certification number: SCXK (Jing): 2016-0002). The rats were housed in the clean level condition animal housing facilities (certification number SYXK (Jing) 2016-0038) of BUCM, at a temperature of 22 ± 1 °C, a humidity of 55 ± 5%, and a 12 h light/dark cycle with free access to tap water and chow. All the protocols were reviewed and approved by the Animal Care Committee of BUCM, China. The animals were allowed to acclimatize for a week while being fed with a standard diet and water ad libitum before experiments. Then they were randomly divided into three groups: sham group (SHAM), ischemic group (MCAO/R), and Kudiezi injection group (KDZI), with 15 rats in each group. In the MCAO/R and KDZI groups, rats were subjected to MCAO/R on the right hemisphere of the brain. The animals in the KDZI group were treated with KDZI injection (4.2 mL/kg i.p. The SHAM and MCAO/R groups received 0.9% sodium chloride at 4.2 mL/kg, i.p.).

### 4.3. Middle Cerebral Artery Occlusion

Rats were anesthetized with 10% chloral hydrate (3.5 mL/kg body weight) intraperitoneally. Cerebral ischemia was conducted according to the method by Longa et al. [36,37] with some modifications. A 2-mm nylon monofilament (Guangzhou Jialing Company, Guangzhou, China) was inserted into the internal carotid artery through a small incision on the common carotid artery and advanced 18 ± 2 mm to occlude the middle cerebral artery, until a slight resistance was felt. Two hours after the induction of ischemia, the filament was slowly withdrawn, and the animals were returned to their cages for a period of 2 h of reperfusion. Throughout the procedure, the body temperature was maintained at 37 °C, with a thermostatically-controlled infrared lamp. Rats in the sham group underwent the same procedure without monofilament insertion.

### 4.4. The Stainings of TTC and H&E

After 7 days of KDZI administration, the rats were anesthetized with 10% chloral hydrate (3.5 mL/kg body weight). Coronal sections of brains (2 mm) were immersed in 1% 2,3,5-triphenyltetrazolium chloride (TTC) in phosphate buffer saline (pH 7.4) at 37 °C for 15 min and then fixed with 10% paraformaldehyde for 10 min [38,39]. The white color represented infarct tissue and the red color represented normal tissue. TTC-stained sections were photographed and the images were analyzed using Image-Pro Plus 6.0 to calculate the infarct area. Hematoxylin and eosin (H and E) staining was conducted according to the protocol of Xiaoli Yan et al. [40]. Images were captured using an Olympus BX53 microscope (Tokyo, Japan) and analyzed using Image-Pro Plus 6.0 to calculate the IOD value.

### 4.5. Plasma Pretreatment and UHPLC-LTQ/Orbitrap Analysis

After the last administration, rats were anesthetized with 10% chloral hydrate (3.5 mL/kg body weight). Blood was collected from the abdominal aorta and blood samples were collected in tubes containing sodium heparin and centrifuged for 3500 rpm, 10 min at 4 °C. The plasma was removed and placed in aliquots, stored at −80 °C until measurement.

Plasma sample preparation was carried out by protein precipitation with acetonitrile. Four-hundred microliters of precipitant (V (acetonitrile): V (methanol) = 3:1) was added to 200 μL of plasma and the mixture was vortexed for 2 min. After 10 min, the mixture was centrifuged at 10,000 rpm for 15 min at 4 °C. Then the supernatant was transferred and dried on a nitrogen blower. Finally, the dried supernatant was re-dissolved in 100 μL mobile phase (A phase 0.1% formic acid aqueous solution). To validate the analytical methodology, pooled QC samples were prepared by mixing all of the samples. Five QC samples were analyzed before sample sequencing, and during the analysis of the sample sequence, one QC sample was run after every five injections [41,42]. Metabolomics analysis was performed on a Thermo Scientific Dionex Utimate 3000 UHPLC Plus Focused coupled to a LTQ/Orbitrap MS system equipped with an electrospray ionization source operating. A 2.1 × 100 mm BEH 1.7 μm C18 column was equipped for all analyses. The measuring method of biomarkers was from reported research [43,25]. The injection volume was 2 μL, the flow rate was 230 μL/min, the column temperature was 30 °C. The mobile phase was a mixture of 0.1% formic acid in water (A) and 0.1% formic acid in acetonitrile (B). Ion source temperature: 350 °C, ionization source voltage: 4 KV, capillary voltage: 35 V, tube lens voltage: 110 V, sheath gas and auxiliary gas are high-purity nitrogen (purity > 99.99%), sheath gas flow rate: 40 arb, the auxiliary air flow rate was 20 arb. Data were acquired using Fourier transform high resolution full sweep (TF, Full scan, Resolution 30000), MS/MS uses data-dependent acquisition, and the broken way is CID. Metabolic profiles were acquired within the range of *m*/*z* 50–1500.

The Proportion of Mobile Phase B Was Optimized as Follows:

Positive ion mode: 0–4 min, 5–7%; 4–5 min, 7–12%; 5–6 min, 12–15%; 6–8 min, 15–30%; 8–17 min 30–48%; 17–21 min, 48–49%; 21–30 min, 49–59%; 30–33 min, 59–61%; 33–35 min, 61–80%; 35–36 min, 80–5%; 36–39 min, 5–5%.

Negative ion mode: 0–3 min, 3–5%; 3–4 min, 5–30%; 4–20 min, 30–36%; 20–21 min, 36–42%; 21–22 min, 42–60%; 22–34 min, 60–64%; 34–39 min, 64–80%; 39–39.1 min, 80–3%; 39.1–42 min, 3–3%.

### 4.6. Data Processing and Analysis

The raw data analysis for plasma samples were processed using the Sieve software package version 2.1 (Thermo Fisher Scientific Inc., San Jose, CA, USA). Before chemometric analysis, the data from each sample were normalized to the sum of the peak area [44]. The pre-processed data were analyzed by principal component analysis (PCA) and quadrature signal correction partial least squares analysis (OPLS), using SIMCA-P13.0 software (Umetrics). Differential Metabolites Identification Used Compound Discoverer 2.0 for spectral matching, combined with Metlin and HMDB databases. The obtained differential components were analyzed by MeV (Multi Experiment Viewer, v4.8, TIGR) for hierarchical clustering analysis and K-mean clustering analysis. All experimental data were expressed as means ± SD, and examined by one-way ANOVA test in the SPSS version 16 software package to calculate the statistical significance. *p*-values < 0.05 were considered significant. Differences in metabolic function and pathway enrichment analysis were using KEGG (http://www.genome.jp/kegg/), MBRole (http://csbg.cnb.csic.es/mbrole2/), and Metpa (http: // Metpa.metabolomics.ca), as well as other online tools.

## 5. Conclusions

In summary, plasma metabolomics analysis of LC-MS provides biomarkers for cerebral ischemia-reperfusion metabolism. Our data shows that amino acid metabolism, energy metabolism, and lipid metabolism are involved in the metabolic pathway of cerebral ischemia and reperfusion. Among them, amino acids are the most important features of cerebral ischemia-reperfusion injury, and are considered to be an important regulator to regulate cell signal transduction. The results also show that taurine and arachidonic acid can be considered as a marker for the diagnosis of clinical diagnostic markers, and LC-MS-based techniques provide a method for the analysis of cerebral ischemia-reperfusion through biological pathways. In the course of the experimental study, further studies are needed to examine the dynamic trends of these biomarkers and the functions of these biomarkers.

## Figures and Tables

**Table 1:** The analytical performance of the UPLC-MS method (*n* = 6).

t_R_-*m/z*	Precision (*RSD*, %)		Stability (*RSD*, %)		Repeatability (*RSD*, %)	
	t_R_	Peak Intensity	t_R_	Peak Intensity	t_R_	Peak Intensity
**Positive**						
2.99–453.34	0.25	3.41	1.88	7.70	0.59	6.51
5.38–215.01	0.08	7.10	0.33	7.28	0.20	6.98
9.89–319.23	0.17	5.80	0.13	3.72	0.17	6.51
14.38–582.38	0.14	5.35	0.23	6.22	0.28	4.52
17.44–255.23	0.035	5.14	0.09	6.89	0.04	5.11
**Negative**						
0.79–269.0	0.65	7.49	0.56	7.07	0.56	6.83
11.33–544.34	0.09	4.99	0.07	4.23	0.09	4.99
12.06–478.34	0.15	2.91	0.14	2.30	0.15	3.45
14.63–341.32	0.11	7.33	0.12	6.31	0.11	8.33
19.42–654.33	0.03	4.94	0.08	5.91	0.08	6.44

Note: t_R_: Retention time.

**Table 2:** Potential plasma biomarkers detected by LC-MS and their intensities in the sham, MCAO/R, and MCAO/R + KDZI groups analyzed by one-way ANOVA.

No.	t_R_	*m/z*	Formula	Identification	Adduct Type	Flod Change	
						MCAO/R/Sham	MCAO/R + KDZI/MCAO/R
1	0.93	118.0856	C_5_H_11_NO_2_	L-Valine	M + H	1.40	0.65
2	1.617	132.1013	C_6_H_13_NO_2_	Isoleucine	M + H	0.68	1.47
3	2.829	166.0856	C_9_H_11_NO_2_	L-Phenylalanine	M + H	0.66	1.45
4	5.51	205.0965	C_11_H_12_N_2_O_2_	L-Tryptophan	M + H	0.81	1.36
5	1.89	192.0655	C_10_H_9_NO_3_	5-Hydroxyindole-3-acetic acid	M + H	1.34	0.64
6	8.585	132.1013	C_6_ H_13_ NO_2_	Leucine	M + H	0.68	1.44
7	28.55	480.28	C_26_H_45_NO_6_S	Taurochenodesoxycholic acid	M - H_2_O - H	0.71	1.43
8	143	283.2623	C_18_H_34_O_2_	Oleic acid	M - H	0.78	1.56
9	33.932	305.2468	C_20_H_32_O_2_	Arachidonic acid	M + H	0.63	1.27
10	35.14	283.2623	C_18_H_36_O_2_	Stearic acid	M - H	0.71	1.39
11	23.119	468.3067	C_22_H_46_NO_7_P	PE (17:0/0:0)	M + H	0.61	1.38
12	26.884	482.3222	C_23_H_48_NO_7_P	LysoPE (0:0/18:0)	M + H	0.81	1.34
13	37.372	482.3221	C_23_H_48_NO_7_P	LysoPE (18:0/0:0)	M + H	0.51	1.31
14	25.379	482.3225	C_23_H_48_NO_7_P	LysoPC (15:0)	M + H	0.61	1.20
15	29.152	496.3376	C_24_H_50_NO_7_P	LysoPC (16:0)	M + H	0.78	1.45
16	33.86	508.3738	C_26_H_54_NO_6_P	LysoPC (p-18:0)	M + H	0.81	1.29
17	34.499	510.3526	C_25_H_52_NO_7_P	LysoPE (0:0/20:0)	M + H	0.74	1.32
18	32.943	510.3526	C_25_H_52_NO_7_P	LysoPC (17:0)	M + H	0.69	1.42
19	24.784	518.3221	C_26_H_48_NO_7_P	LysoPC (18:3(6z, 9z, 12z))	M + H	0.89	1.23
20	29.081	518.32	C_26_H_48_NO_7_P	PC(18:3(9z, 12z, 15z)/0:0)	M + H	0.82	1.42
21	31.039	522.3535	C_26_H_52_NO_7_P	LysoPC (18:1(9z))	M + H	0.71	1.28
22	33.776	522.3535	C_26_H_52_NO_7_P	LysoPC (18:1(11z))	M + H	0.63	1.32
23	15.814	536.333	C_27_H_54_NO_7_P	LysoPE (0:0/22:1(13z))	M + H	0.89	1.41
24	17.288	536.3331	C_27_H_54_NO_7_P	LysoPE (22:1(13z)/0:0)	M + H	0.71	1.24
25	35.514	536.3695	C_27_H_54_NO_7_P	LysoPE (0:0/22:1(13Z))	M + H	0.83	1.42
26	32.715	538.3848	C_27_H_56_NO_7_P	LysoPE (0:0/22:0)	M + H	0.79	1.23
27	30.365	538.3858	C_27_H_56_NO_7_P	LysoPE (22:0/0:0)	M + H	0.70	1.32
28	24.365	542.3221	C_28_H_48_NO_7_P	LysoPC (20:5(5z, 8z, 11z, 14z, 17z))	M + H	0.61	1.33
29	30.525	546.3532	C_28_H_52_NO_7_P	LysoPC (20:3(5z, 8z, 11z))	M + H	0.74	1.34
30	29.248	546.3534	C_28_H_52_NO_7_P	LysoPC (20:3(8z, 11z, 14z))	M + H	0.82	1.31
31	34.412	548.369	C_28_H_54_NO_7_P	LysoPC (20:2(11z, 14z))	M + H	0.82	1.34
32	29.521	570.3532	C_30_H_52_NO_7_P	LysoPC (22:5(7z, 10z, 13z, 16z, 19z))	M + H	0.81	1.34

Note: t_R_: Retention time.

**Table 3:** The information of the relevant metabolic pathways.

Pathyway Name	In Set	*p*-Value (×10^3^)	*FDR* Correction (×10^3^)
Tryptophan metabolism	2	0	0
Fatty acid elongation unsaturated	3	0.00000296	0.0000266
Biosynthesis of unsaturated fatty acids	3	0.00000723	0.000803
Valine, leucine and isoleucine degradation	3	0.0000744	0.00156
Valine, leucine and isoleucine biosynthesis	3	0.000422	0.00814
Aminoacyl-tRNA biosynthesis	4	0.00119	0.044
Biosynthesis of secondary metabolites	5	0.00157	0.0173
Glucosinolate biosynthesis	5	0.0332	0.38
Metabolic pathways	3	0.116	0.522
jasmonoyl-amino acid conjugates biosynthesis I	4	3.29	7.4
jasmonoyl-amino acid conjugates biosynthesis II	3	6.53	28.3
